# First-Principles Computational Study of the Modification Mechanism of Graphene/Graphene Oxide on Hydroxyapatite

**DOI:** 10.3390/ma15238652

**Published:** 2022-12-05

**Authors:** Yanqing Wang, Minghui Xie, Zheng Zhou, Muhammad Junaid, Weilin Zong, Shengyang Du

**Affiliations:** 1 School of Materials and Physics, China University of Mining and Technology, Xuzhou 221116, China; 2 School of Electrical Engineering, China University of Mining and Technology, Xuzhou 221116, China; 3 Department of Orthopedics, Affiliated Hospital of China University of Mining and Technology, Xuzhou 221116, China

**Keywords:** composite material system, first-principles calculation, density of states, differential charge density, mechanical property

## Abstract

Due to its unique crystal structure and nano-properties, hydroxyapatite (HA) has become an important inorganic material with broad development prospects in electrical materials, for fire resistance and insulation, and in bone repair. However, its application is limited to some extent because of its low strength, brittleness and other shortcomings. Graphene (G) and its derivative graphene oxide (GO) are well known for their excellent mechanical properties, and are widely used to modify HA by domestic and foreign scholars, who expect to achieve better reinforcement and toughening effects. However, the enhancement mechanism has not been made clear. Accordingly, in this study, G and GO were selected to modify HA using the first-principles calculation method to explore the theory of interfacial bonding of composites and explain the microscopic mechanism of interfacial bonding. First-principles calculation is a powerful tool used to solve experimental and theoretical problems and predict the structure and properties of new materials with precise control at the atomic level. Therefore, the bonding behaviors of hydroxyapatite (100), (110) and (111) crystal planes with G or GO were comprehensively and systematically studied using first-principles calculation; this included analyses of the density of states and differential charge density, and calculations of interfacial adhesion work and elastic moduli. Compared to HA (100) and (111) crystal planes, HA (110) had the best bonding performance with G and with GO, as revealed by the calculation results. The composite material systems of HA (110)/G and HA (110)/GO had the smallest density of states at the Fermi level, the largest charge transfers of Ca atoms, the largest interfacial adhesion work and the most outstanding elastic moduli. These results provide a theoretical basis for the modification of HA to a certain extent, and are beneficial to the expansion of the scope of its application.

## 1. Introduction

As an important component of human bone, HA is well known in the field of biomaterial as having good biocompatibility and biological activity [1,2,3]. It can produce violent chemical bonds with human bone and promote the growth of new bone. Moreover, due to the nature of its wide band gap (3.577 eV) and difficult electrons transition, HA has recieved increasing attention in the field of insulation material, either as the main material or as an additive material [4]. For example, Zhao [5] prepared HA whiskers modified using ethylene glycol, and then made PEG@HANW nanocomposite insulation paper. HA’s surface also has multiple hydroxyl groups, which can provide sufficient reaction sites for in situ modification [6], and outstanding electrolyte wettability and liquid retention [7]. Therefore, HA has attracted increasing attention in the fields of thermal insulation materials, refractory paper, biomedical materials and high strength nanocomposites [8,9,10]. However, HA has the disadvantages of high brittleness and low strength [11,12,13,14], which limit its application to some extent. Accordingly, how to improve the comprehensive performance of HA is the key to solving its limited use as an important ceramic material. Researchers have conducted a wide range of studies addressing this issue. Wu [15] found that nano-HA ceramics could decrease the hardness and fracture toughness of HA ceramics with an increase in crystallinity. First-principles and molecular dynamics simulations were used by Shang [16] to study HA’s crystal structure and adsorption capacity for macromolecular drugs; it was found that Zn-doping effectively improved the binding energy between doxorubicin (DOX) and HA. Yuan [17] used molecular dynamics to study the compounding of (001), (100) and (110) crystal planes of Zn-doped HA with PLA, and analyzed their mechanical properties. It was found that Zn-doping decreased the elastic properties of the composite material systems, and that the elastic properties of the HA (110) crystal plane were better than those of (001) and (100) crystal planes.

Carbon-based materials are abundant in the earth and easy to obtain. They also have good chemical stability and diverse structures, such as one-dimensional carbon nanotubes, two-dimensional graphene and its derivatives, etc. [18,19]. Therefore, carbon-based materials have become a research hotspot at home and abroad in recent years. G and GO, in particular, have been studied extensively. Possessing high strength and good toughness, G is a monolayer material with a two-dimensional honeycomb crystal structure formed by tightly packed carbon atoms [20,21,22], which has garnered considerable research attention. Kakanakova-Georgieva [23] prepared 2D InO using a robust metal organic chemical vapor deposition (MOCVD) through the directional intercalation and deposition kinetics of indium atoms at the G/SiC interface. Lundgren [24] employed ab initio molecular dynamics (AIMD) simulations to assess graphene’s thermal stability, mechanical properties (such as pristine 2D InBi and H-InBi (hydrogenated 2D InBi)), and 2D InBi heterostructures. GO is a derivative of graphene with functional groups of hydroxyl and epoxy groups covering the surface of a single layer of carbon atoms [25,26]. GO and G have many similar characteristics, but also have differences. For example, due to the oxygen-containing functional groups in GO, mutual repulsion between different layers is inevitably produced, which makes its dispersion, combination and compatibility better than that of G. Studies [27] have shown that Escherichia coli dies when being cultured on a surface of G, which indicates that G has certain antibacterial properties. These properties greatly benefit HA/G system biomaterials used in bone graft and bone repair applications and as insulation in items such as gloves and masks. Therefore, in this study, G and GO were selected to modify HA. The interface bonding state and mechanical properties of the composite materials were calculated and analyzed using the computational simulation method to provide some theoretical support for improving the comprehensive performance and application expansion of HA. Hassan Nosrati [28] explored the effect of diethylene glycol on the synthesis of HA/GO and found that the presence of diethylene glycol reduced the average grain size of HA from 26 nm to 16 nm, improved the reduction rate of GO and increased the elastic modulus, hardness and fracture toughness of the composite. Zhang [29] and Liu [30] prepared a (graphene nanosheet) GNS/HA composite via plasma sintering, and found that the addition of GNS tremendously improved the mechanical properties of HA, and effectively prevented the growth of HA during high-temperature treatment, which played an immense role in toughening HA. Fan [31] prepared GNS/HA nano-rod composites using the one-pot hydrothermal method and found that this method effectively improved the hardness and Young’s modulus of HA. The above research showed that the addition of G or GO could improve the mechanical properties of HA. However, these results were only explained from the macroscopic perspective using experimental means; the microscopic mechanism of the improvement remains unclear and unsupported by numerical study. First-principles is a calculation method with a perfect theoretical basis; it can not only accurately calculate the structure, properties and energy of materials, but also deeply explore the physical and chemical properties of surfaces and interfaces. Furthermore, the structural properties of materials calculated using this method are similar to the actual properties; thus, first-principles calculation has become a key technology in computational materials science. Therefore, in this study, the first-principles calculation method was used to numerically calculate and try to explain the composite’s mechanical properties improvement mechanism of HA modified using G or GO, and to explore the best combination surface of G or GO and HA. Notably, G and GO have good conductivity; therefore, in this study, the additional amount of G or GO was well controlled to modify HA’s mechanical properties, but not its insulation properties.

## 2. Calculation Method and Model

### 2.1. Calculation Method

In this study, calculations of state and differential charge densities, as well as energy, were carried out using the plane wave program of the functional module of CASTEP (Cambridge Sequential Total Energy Package) in Materials Studio software. Calculation parameters were set as follows. Exchange-correlation was represented using the RPBE (Revised-Perdew-Burke-Ernzerhof) function in the generalized gradient approximation (GGA). The Kohn–Sham equation was solved self-consistently. The Gaussian expansion was adopted using an expansion width of 0.05 eV. The atomic force exerted on each atom was set less than 0.01 eV to ensure full relaxation of each atom in the crystal structure cell. The interaction between the ionic core and valence electrons was described using the ultra-soft pseudopotential. The total energy convergence value of the system was set to 2 × 10^−6^ eV/atom with crystal structure cell optimization using the BFGS algorithm. The cutoff energy and the K point were set as 420 eV and 3 × 3 × 1, respectively.

### 2.2. Establishment of Model

**Figure 1:**
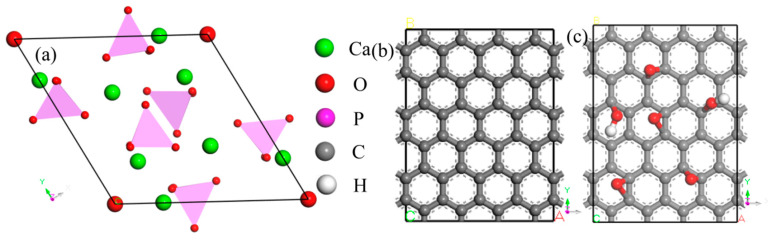
The models of (**a**) HA, (**b**) G, (**c**) GO.

**Figure 2:**
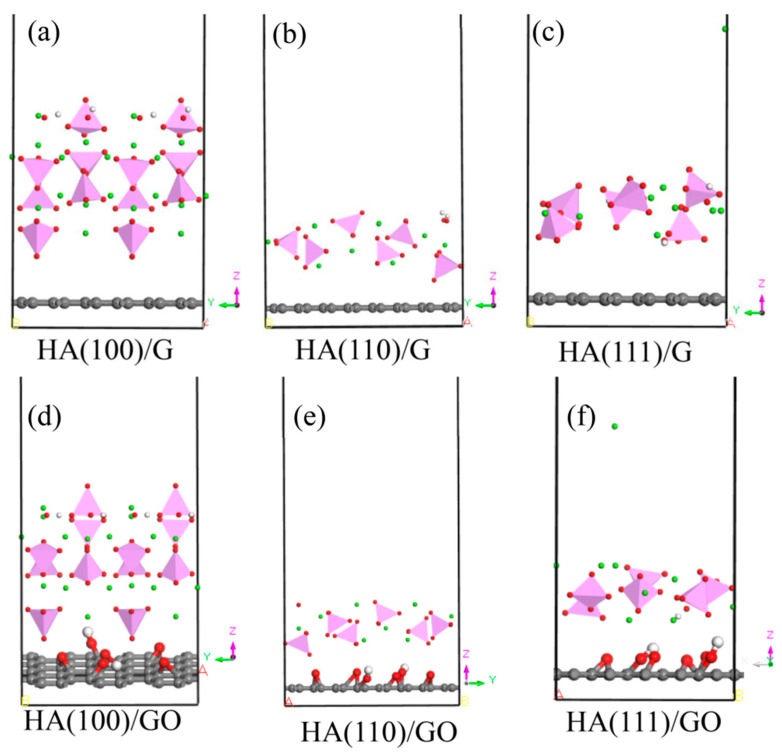
Supercrystal cell modes of HA with G and with GO in different crystal planes (the atom colors are the same as in Figure 1. (**a**) HA(100)/G, (**b**) HA(110)/G, (**c**) HA(111)/G, (**d**) HA(100)/GO, (**e**) HA(110)/GO, (**f**) HA(111)/GO.

HA, Ca_10_(PO_4_)_6_(OH)_2_, belongs to a hexagonal system with a space group of p63/m. G is a structure with closely packed SP2 hybrid and two-dimensional honeycomb carbon atoms. GO is a structure grafted with functional groups, such as hydroxyl and epoxy, based on G. The structural models of HA, G and GO are shown in Figure 1. Three crystal planes, (100), (110) and (111), were cut for HA, and six supercells containing the minimum number of atoms were established using the above three crystal planes and G or GO, as shown in Figure 2. In order to achieve the most effective nucleation, the lattice mismatch was set to less than 6% in the calculation according to the theory of lattice mismatch [32,33].

## 3. Discussion and Analysis of Results

### 3.1. Density of States

**Figure 3:**
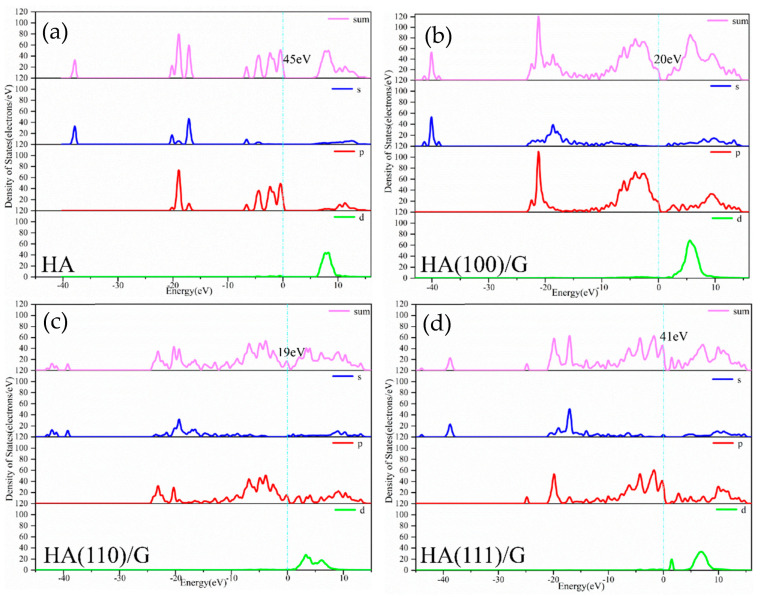
DOS diagrams of pure HA and G modified HA composite systems: (**a**) HA, (**b**) HA(100)/G, (**c**) HA(110)/G, (**d**) HA(111)/G.

**Figure 4:**
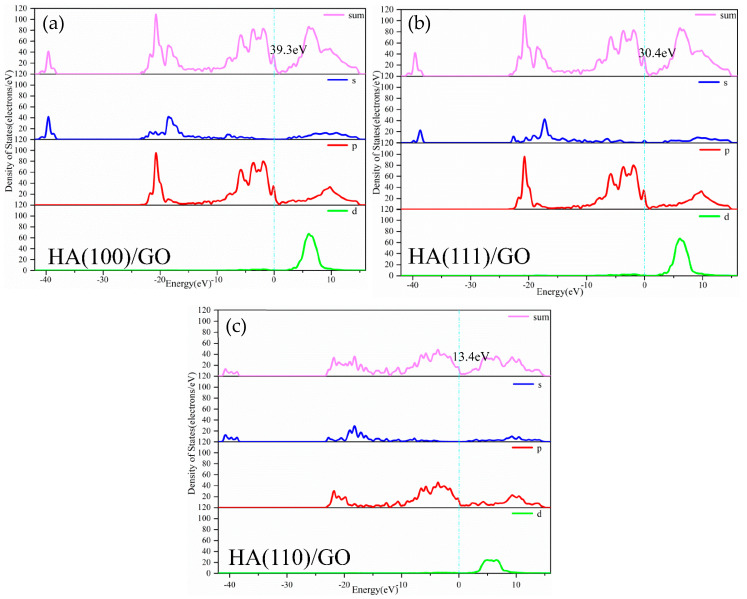
DOS diagrams of GO modified HA composite systems: (**a**) HA(100)/GO, (**b**) HA(110)/GO, (**c**) HA(111)/GO.

As the density of electronic energy states per energy interval unit in the material system, the density of states (DOS) facilitates understanding and assessment of the electronic orbit, energy state, property changes and stability of a composite material system to a certain extent. The total DOS and partial DOS of the above six supercells with different material systems were calculated, curves were obtained, and the results for pure HA were taken for comparison, as shown in Figure 3 and Figure 4, respectively.

Pure HA’s conduction band energy level was continuously distributed from 5~16 eV; however, its valence band energy levels were discretely distributed from −39~0 eV, and its DOS value at the Fermi level was 45 eV, which was primarily contributed via p orbitals, as shown in Figure 3a. After the HA (100), (110) and (111) crystal planes were modified using G, energy level distribution ranges, DOS values at the Fermi level and orbital contributions changed. The HA (100)/G material system’s conduction band level was continuously distributed from 0~15 eV, its valence band levels were discretely distributed from −42~0 eV, and its DOS value at the Fermi level was 20 eV, which was primarily contributed via p orbital, as shown in Figure 3b. The HA (110)/G material system’s conduction band energy level was distributed from 0~15 eV, its valence band energy levels were discretely distributed from −44~0 eV, and its DOS value at the Fermi level was 19 eV, which was contributed via s and p orbitals (the p orbital was dominant), as shown in Figure 3c. The HA (111)/G material system’s conduction band energy level was continuously distributed from 0~16 eV, its valence band energy levels were discretely distributed from −44~0 eV, and its DOS value at the Fermi level was 41 eV, which was contributed via s and p orbitals (the p orbital was dominant), as shown in Figure 3d. Comprehensive comparison and analysis of the above results indicated that, for the three composite material systems modified using G, in either the conduction band or the energy band the energy level distribution range covered the pure HA value and widened. In addition, DOS values at the Fermi level were all primarily contributed via p orbitals, as was the case with pure HA. This indicates that the basic properties of HA can be maintained in the composite material systems modification using G. However, it should be noted that small and continuous characteristic peaks increased, and DOS values at the Fermi level decreased, which indicates that the composite material systems generated new energy levels and became more stable. The HA (110)/GT material system had the smallest DOS value (19 eV) at the Fermi level, which indicates that the HA (110)/G material system was the most stable, and that it was easier to combine G with the crystal plane (110) of HA.

After HA (100), (110) and (111) crystal planes were modified using GO, energy level distribution ranges, DOS values at the Fermi level and orbital contributions also changed. The HA (100)/GO material system’s conduction band level was continuously distributed from 0~16 eV, its valence band levels were discretely distributed from −41~0 eV, and its DOS value at the Fermi level was 30.3 eV, which was primarily contributed via p orbital, as shown in Figure 4a. The HA (110)/GO material system’s conduction band energy level was distributed from 0~15 eV, its valence band energy levels were discretely distributed from −41~0 eV, and its DOS value at the Fermi level was 13.4 eV, which was primarily contributed via p orbitals, as shown in Figure 4b. The HA (111)/GO material system’s conduction band energy level was continuously distributed from 0~15 eV, its valence band energy levels were discretely distributed from −40~0 eV, and its DOS value at the Fermi level was 30.3 eV, which was contributed via s and p orbitals (the p orbital was primarily dominant), as shown in Figure 4c. After modification using GO, the same rules as those of modification using G were observed. First, the basic properties of HA could be maintained in HA/GO composite material systems, which was consistent with the literature [34]; second, new energy levels were generated in HA/GO composite material systems, and these material systems were more stable. Among them, the HA (110)/GO material system had the smallest DOS value (13.4 eV) at the Fermi level, which indicates that the HA (110)/GO material system was the most stable, and that it was easier to combine GO with the crystal plane (110) of HA.

### 3.2. Differential Charge Density

**Figure 5:**
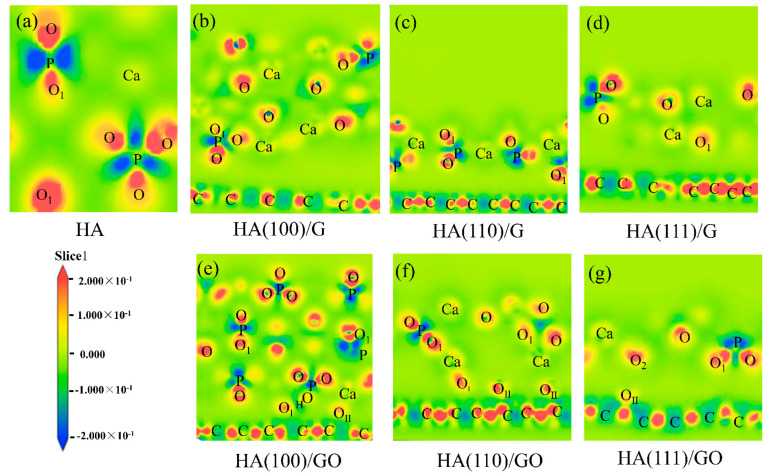
DCD maps of (**a**) pure HA and composite systems, (**b**) HA (100)/G, (**c**) HA (110)/G, (**d**) HA (111)/G, (**e**) HA (100)/GO, (**f**) HA (110)/GO and (**g**) HA (111)/GO.

The differential charge density (DCD) clearly and intuitively shows the aggregation and distribution trend of each atomic charge in a material system. Through the analysis of the DCD map of a composite material system, charge transfer and bonding types in that material system can be clearly explained. The DCD maps of the above six composite material systems were obtained; the map of pure HA was obtained for comparison. The DCD maps reveal all the atomic positions in the material systems, and express gains or losses of electrons in general, with red representing gains of electrons and blue representing losses of electrons, as shown in Figure 5. Among all the atoms, different types of O atoms were marked for distinction. The O atom in a P−O single bond in HA was labeled as O, the O atom in a P−O double bond in HA was labeled as O_1_, the O atom in hydroxyl was labeled as O_2_, the O atom in the hydroxyl group of GO was labeled as O_I_, and the O atom in the epoxy group of GO was labeled as O_II_.

In the DCD map of pure HA, the P atom lost electrons, the O atom gained electrons, and the Ca atom lost a small number of electrons; this was consistent with the nature of each atom in HA, as shown in Figure 5a. However, whether the HA (100), (110) and (111) crystal planes were modified using G or GO, a charge transfer occurred between them, and the electron cloud distributions of HA consequently changed; however, there were no obvious electron cloud overlaps between them, which indicates that they were combined via Van der Waals force [35]. In the G area, a strong electron gain region between C−C atoms was observed, and a large number of electrons were shared, which is characteristic of a typical covalent bond [36]. Among the six composite material systems, HA (110)/G and HA (110)/GO were the best combinations, because their charge transfers and electron cloud distributions showed the most significant changes. Of the three atoms P, O and Ca in HA, Ca atoms generated the greatest charge transfer; however, the nature of their gain or loss of electrons did not change, that is, some properties of HA remained, as shown in Figure 5b–g.

### 3.3. Interface Adhesion Work

Interfacial adhesion work is an important measurement parameter for interfacial bonding performance, which is an expression of the work performed by the outside world when the surface per unit area has adhered to the interface; in this study, it refers to measurements of the interaction between G and HA or GO and HA. The greater the interfacial adhesion work, the better the bonding property of the interface. The work of adhesion Wad of this AB model for HA/G can be expressed as [37]:

(1) $$Wad=\frac{E_{HA}^{slab}+E_{G}^{slab}-E_{HA/G}^{}}{A}$$

where $E_{HA}^{slab}$ is the energy of HA when the free interface is constituted, $E_{G}^{slab}$ is the energy of G when the free interface is constituted, $E_{HA/G}^{}$ is the energy of the composite material system, and A is the interfacial area of the composite material system. The work of adhesion Wad of HA/GO can be similarly expressed. The relevant index values and calculation results of interface adhesion work for each system are shown in Table 1.

The interfacial adhesion work of the six composite material systems were all positive, which indicates that G and GO can theoretically combine with the (100), (110) and (111) crystal planes of HA. However, the bonding properties of HA (110) with G and GO were better than those of the (100) and (111) crystal planes because of its relatively higher adhesion work values of 0.691 J/m^2^ (G) and 1.041 J/m^2^ (GO).

### 3.4. Elastic Modulus

Elastic modulus is a manifestation of the binding force between microcosmic atoms in crystals [38]. Macroscopically, it is the ability of materials to resist external pressure within a certain range, and it is an important mechanical index of materials. Under the same pressure, the smaller the elastic modulus is, the more serious the deformation of the material is, and vice versa. Bulk modulus and shear modulus are the key indicators that characterize the fracture strength and stiffness of materials, respectively. The greater the value, the higher the stiffness of the material. Young’s modulus characterizes the tensile resistance of materials [39]. Three elastic modulus index values for each system and pure HA were calculated, as shown in Table 2. With modifications using G, the three elastic modulus index values for HA (100)/G, HA (110)/G and HA (111)/G composite material systems all improved; maximum improvement was observed for the HA (110)/G composite material system. However, with modifications using GO, only the HA (110)/GO composite material system’s three elastic moduli improved. That is, the elastic moduli properties of HA (110)/G and HA (110)/GO were better than those of the (100) and (111) crystal planes. Table 2 also shows that the mechanical properties of HA modified using G were better than those modified using GO, which was consistent with the experimental results that the mechanical properties of GO were much lower than those of G due to the destruction of carbon skeleton during the oxidation process [40].

## 4. Conclusions

Three HA crystal planes, (100), (110) and (111), were interface modified using G and using GO. Using first-principles calculation and analysis, comprehensive calculations of DOS, DCD, interface adhesion work and elastic modulus for the six composite material systems and pure HA were conducted. The results show that HA (110)/G and HA (110)/GO material systems had the lowest DOS values at the Fermi level, the most obvious charge transfers, the largest interface adhesion work and maximum improvements in elastic moduli compared with HA (100) and (111) crystal planes; the HA (110) crystal plane combined best with G and with GO. Compared with the other composites, HA (110)/G and HA (110)/GO composite material systems had the best bonding properties and elastic modulus properties. Therefore, this finding provides some theoretical guidance for the future design of HA and G and HA and GO composite systems and expands HA’s application range. 

## Figures and Tables

**Table 1:** Related index values and calculation results of interface adhesion work for each system.

	$E_{HA}^{slab}$/eV	$E_{G}^{slab}$/eV	$E_{HA/G}^{}$/eV	A/nm^2^	Wad/J/m^2^
HA (100)/G	−45,683.534	−75,52.622	−53,239.145	1.289	0.371
HA (110)/G	−22,551.869	−7450.200	−30,007.251	1.200	0.691
HA (111)/G	−22,553.747	−9310.013	−31,865.209	1.479	0.157
HA (100)/GO	−45,126.202	−10,085.343	−55,219.135	1.289	0.942
HA (110)/GO	−22,556.637	−8768.421	−31,332.868	1.200	1.041
HA (111)/GO	−22,548.922	−11,948.078	−34,502.087	1.479	0.678

**Table 2:** Calculation results of three elastic modulus index values for each system.

	Bulk Modulus (B)/GPa	Shear Modulus (G)/GPa	Young’s Modulus (E)/GPa
HA	59.54	31.01	78.12
HA (100)/G	134.18	47.88	264.17
HA (110)/G	138.07	49.28	279.83
HA (111)/G	69.86	33.34	185.87
HA (100)/GO	57.20	31.88	130.58
HA (110)/GO	68.76	32.59	139.48
HA (111)/GO	56.37	30.49	134.54

## Data Availability

The study did not report any data.
